# Current status of precision oncology in adult glioblastoma

**DOI:** 10.1002/1878-0261.13678

**Published:** 2024-06-20

**Authors:** Johannes Weller, Anna‐Laura Potthoff, Thomas Zeyen, Christina Schaub, Cathrina Duffy, Matthias Schneider, Ulrich Herrlinger

**Affiliations:** ^1^ Department of Neurooncology, Center for Neurology University Hospital Bonn Germany; ^2^ Department of Neurosurgery University Hospital Bonn Germany

**Keywords:** biomarker, glioblastoma, molecular profiling, personalized medicine, precision oncology, targeted treatment

## Abstract

The concept of precision oncology, the application of targeted drugs based on comprehensive molecular profiling, has revolutionized treatment strategies in oncology. This review summarizes the current status of precision oncology in glioblastoma (GBM), the most common and aggressive primary brain tumor in adults with a median survival below 2 years. Targeted treatments without prior target verification have consistently failed. Patients with BRAF V600E‐mutated GBM benefit from BRAF/MEK‐inhibition, whereas targeting EGFR alterations was unsuccessful due to poor tumor penetration, tumor cell heterogeneity, and pathway redundancies. Systematic screening for actionable molecular alterations resulted in low rates (< 10%) of targeted treatments. Efficacy was observed in one‐third and currently appears to be limited to BRAF‐, VEGFR‐, and mTOR‐directed treatments. Advancing precision oncology for GBM requires consideration of pathways instead of single alterations, new trial concepts enabling rapid and adaptive drug evaluation, a focus on drugs with sufficient bioavailability in the CNS, and the extension of target discovery and validation to the tumor microenvironment, tumor cell networks, and their interaction with immune cells and neurons.

## Graphical abstract

This review summarizes the current status of precision oncology – targeted therapies based on individual genetic profiles – in glioblastoma. Although some treatments show promise, their applicability and effectiveness remain limited, underlining the need for innovative approaches. Novel concepts to advance precision oncology and improve patient outcomes are discussed.

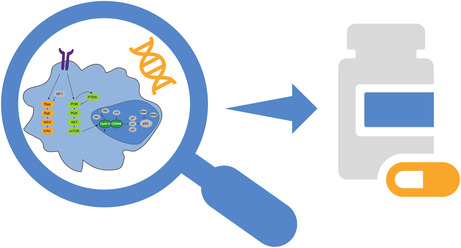

**Abbreviations**
ALK: anaplastic lymphoma kinase
AMPAR: α‐amino‐3‐hydroxy‐5‐methyl‐4‐isoxazoleproprionic acid receptor
CAR: chimeric antigen receptor
CCNU: lomustine
CD95L: CD95 ligand
CDK4/6: cyclin‐dependent kinase 4/6
CDKN2A: cyclin‐dependent kinase inhibitor 2A
CNS: central nervous system
CSF‐R1: colony‐stimulating factor 1 receptor
CXCL12/SDF1: CXC chemokine ligand 12/stromal cell derived factor 1
CXCR4: CXC chemokine receptor 4
EGFR: epidermal growth factor receptor
EGFRvIII: EGFR variant III
EV: extracellular vesicle
FGFR: fibroblast growth factor receptor
FLT: FMS‐like tyrosine kinase, member of VEGFR family
GBM: glioblastoma
HER2: human epidermal growth factor receptor 2
HGF/SF: hepatocyte growth factor/scatter factor
IDH: isocitrate dehydrogenase
IL13R: interleukin 13 receptor
IL4R: interleukin 4 receptor
MDM2: mouse double minute 2 homolog
MDM4: mouse double minute 4 homolog
MEK: mitogen‐activated protein kinase kinase
MGMT: O^6^‐methylguanine‐DNA‐methytransferase
mTOR: mammalian target of rapamycin
NF1: neurofibromin 1
NGS: next‐generation sequencing
NSCLC: non‐small‐cell lung cancer
NTRK: neurotrophic tropomyosin receptor kinase
ORR: objective response rate
OS: overall survival
PARP: polyADP‐ribose‐polymerase enzymes
PD‐1: programmed cell death protein 1
PDGFR: platelet‐derived growth factor receptor
PD‐L1: programmed death‐ligand 1
PFS: progression‐free survival
PFS2/PFS1: ratio of 2nd PFS/1st PFS
PI3K: phosphatidylinositol 3‐kinase
pSTAT3: phosphorylated signal transducer and activator of transcription 3
PTEN: phosphatase and tensin homolog
RTK: receptor tyrosine kinase
TGFβ: transforming growth factor β
TKI: tyrosine kinase inhibitor
TME: tumor‐associated microenvironment
TMZ: temozolomide
VEGFR: vascular endothelial growth factor receptor

## 1 Introduction

In recent years, there has been a rapidly expanding amount of information on the molecular vulnerabilities of cancer cells, informing the development and application of targeted drugs. Actionable targets may comprise the genomic, genetic, epigenetic, transcriptional, proteomic, and metabolomic properties of tumor cells and may vary from one individual tumor to the next. Early studies of targeted treatment determined target engagement and therapeutic efficacy in *post hoc* subgroup analyses of larger non‐selective and target‐agnostic trials. Precision oncology goes in the opposite way, analyzing an individual tumor to inform treatment tailored to its biological characteristics [1, 2]. Target detection is a prerequisite, but the decision to apply a drug to an individual patient also requires antitumor efficacy based on – at minimum – biological plausibility, key insights from preclinical research, or clinical data from other tumor types or optimally the same tumor entity harboring the targeted vulnerability. Several classifications formalize the grading of clinical significance and actionability of molecular targets [3, 4, 5].

Recent clinical trials, including molecularly targeted agents and immunotherapies, have achieved unprecedented survival in solid cancers enriched with specific molecular alterations. The cancer types that have benefited the most are molecularly defined subgroups of melanoma, non‐small cell lung cancer (NSCLC), and breast cancer. In melanoma, high response rates (> 60%) and prolonged progression‐free survival (PFS) and overall survival (OS) were achieved [6, 7, 8, 9, 10]. In NSCLC, targeted treatment based on defined molecular alterations became the standard of care for first‐line therapy [11]. In breast cancer, targeted treatment is also well established, and several compounds are available and sequentially applied [12, 13, 14, 15, 16, 17]. Targeted treatment is increasingly established for other cancer entities [18, 19, 20, 21, 22]. Of note, genetic tumor cell vulnerabilities are not restricted to a single cancer entity but may occur in many different entities, albeit at a low percentage. This opens the door for the application of drugs registered for one entity to be used for tumors from other cancer entities that harbor the actionable alteration, e.g. in NSCLC and gliomas [23, 24]. Accordingly, entity‐agnostic clinical trials and drug registrations focusing on the presence of a particular alteration have been initiated [25]. However, cross‐entity comparisons illustrate that the response to a certain compound may vary significantly, owing to the differential activation of concurrent pathways, the occurrence of different mutations, and the differing tissue permeability for the respective drug [26].

This review explores the current status and perspective of precision oncology in isocitrate dehydrogenase (IDH) wildtype glioblastoma (GBM), the most common and aggressive malignant CNS tumor in adults.

## 2 GBM therapy: current status and potential therapeutic targets

### 2.1 Current standard of care in the newly diagnosed and progressive setting

Maximum‐safe tumor resection [27], radiotherapy of the tumor region [28, 29], and concomitant and adjuvant temozolomide (TMZ) [29], an alkylating agent with high CNS penetrance and bioavailability, represent the current standard of care for first‐line GBM treatment [30]. Tumor treating fields applying alternating electrical fields through the scalp are an additional treatment option [31]. Furthermore, nitrosoureas such as lomustine (CCNU), also an alkylating agent with similarly favorable CNS penetrance, are available for first‐ or further‐line treatment [32, 33]. In some countries, the anti‐vascular endothelial growth factor (VEGF) A antibody bevacizumab is used as a non‐selective second‐line therapy, although it prolongs only PFS but not OS [34]. The portfolio of further line therapies is enriched only by re‐resection [35], re‐irradiation [36], and possibly the multi‐kinase inhibitor regorafenib (see Section 3.1 [37]), while the aggressive course of disease limits the time for further treatment lines. Thus, GBM patients need new and effective treatment options that should ideally be guided by predictive molecular characteristics to ensure that medications with a high likelihood of effectiveness are applied.

### 2.2 Molecular predictors of therapeutic benefit from alkylating chemotherapy

O^6^‐Methylguanine‐DNA‐methytransferase (MGMT) is a DNA repair enzyme that confers resistance to alkylating chemotherapy. Its expression is mainly regulated by epigenetic modification, and the *MGMT* gene promoter methylation status was introduced more than 15 years ago as a prognostic and predictive factor strongly associated with benefit from TMZ and CCNU [38, 39]. Patients with an *MGMT* promoter‐methylated (MGMT‐methylated) GBM receiving TMZ had a significantly longer median OS of up to 31.4 months [33, 39, 40, 41] compared to approximately 17 months in patients without *MGMT* promoter methylation (*MGMT*‐unmethylated) [42]. As some benefit from TMZ cannot be excluded in patients with an unmethylated *MGMT* promoter [39], it is accepted that the *MGMT* promoter methylation status is not a prerequisite for applying TMZ in GBM first‐line treatment. This notion has some notable exceptions, indicating the first step toward a molecularly informed treatment of GBM. First, in patients > 65 years with an *MGMT*‐unmethylated GBM, TMZ showed just under no significant benefit (*P* = 0.055), and therefore, radiotherapy alone is a valid therapeutic option in this subgroup [43]. The low activity of TMZ in patients with *MGMT*‐unmethylated GBM prompted selective clinical trials for this subgroup, allowing the comparison of experimental treatment arms to placebo without TMZ in the standard arm [42, 44]. Second, the CeTeG/NOA‐09 phase III trial demonstrated an increased median OS of 48.1 months in patients with *MGMT*‐methylated GBM receiving CCNU/TMZ, rendering this combined chemotherapy a treatment option selectively for patients belonging to this molecularly defined subgroup [33].

Beyond *MGMT* gene promoter methylation analysis, the subclassification of brain tumors in general and GBM in particular into DNA methylation‐based subgroups has spawned hopes to identify further treatment‐guiding predictive patterns [45]. The RTK II subgroup is enriched for epidermal growth factor receptor (EGFR) amplification and chromosome 10 loss, which corresponds to the ‘classic’ gene expression subtype described by Verhaak et al. [46]. The RTK I subgroup is characterized by platelet‐derived growth factor (PDGFR) A amplification, corresponding to the ‘proneural’ expression subtype. The MES subgroup (‘mesenchymal’ expression subtype) frequently bears NF1 and PTEN alterations [46, 47, 48]. Comparing radiotherapy versus TMZ in elderly GBM patients, the NOA‐08 trial found the prognostic impact of *MGMT* promoter methylation status was limited to GBM of the RTK II subgroup and was absent in the RTK I and MES subgroups.

In addition, a biosimulation study predicted individual differential responses to CCNU/TMZ and TMZ treatments [49]. These results require prospective validation and are met with skepticism because, despite a common genetic background, GBM cell states show considerable plasticity [50, 51].

### 2.3 Genetic vulnerabilities and potential treatment targets in GBM

**Fig. 1:**
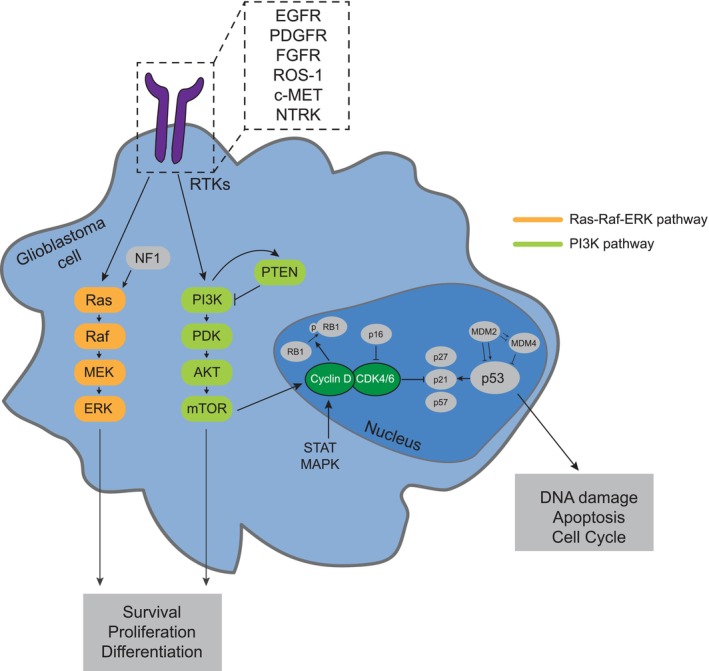
Frequent targetable alterations in adult IDH wildtype glioblastoma. This figure illustrates a selection of currently targetable frequent molecular alterations in glioblastoma. Receptor tyrosine kinases (RTKs) are among the most frequent alterations. Following RTK activation, downstream signal transduction involves the phosphatidylinositol 3‐kinase (PI3K) pathway with frequent PI3K or phosphatase and tensin homolog (PTEN) alterations, and the Ras pathway with frequent activation via neurofibromin 1 (NF1) mutations. Cell cycle dysregulation involving cyclin‐dependent kinase pathway alterations occurs in 90% of glioblastoma, frequently involving p16 (encoded by cyclin‐dependent kinase inhibitor 2A/B [CDKN2A/B]), cyclin‐dependent kinase 4/6 (CDK4/6), p52, mouse double minute 2/4 homolog (MDM2/4), or RB1. For detailed explanations, refer to Section 2.3 in the main text. AKT, anaplastic lymphoma kinase; EGFR, epidermal growth factor receptor; FGFR, fibroblast growth factor receptor; IDH, isocitrate dehydrogenase; MAPK, mitogen‐activated protein kinase; mTOR, mammalian target of rapamycin; NTRK, neurotropic tropomyosin receptor kinase; PDGFR, platelet‐derived growth factor receptor; PDK, 3′‐phosphoinositide‐dependent kinase; STAT, signal transducer and activator of transcription.

The landscape of genetic alterations in GBM is well known, and clinically annotated expression and mutation data are readily available from TCGA and other data repositories [46, 52]. As shown in Fig. 1, genetic alterations in GBM frequently involve:

1. Alterations in growth factor receptors/receptor tyrosine kinases (RTK). The most frequent example is EGFR, altered by approximately 60% [52]. This includes amplification in about 40% [52, 53], frequently associated with other alterations such as EGFR mutations or deletions, the most important being the EGFR variant III (EGFRvIII). While PDGFR is also frequently altered (10–15%), further alterations, including fibroblast growth factor receptor (FGFR, 2–5%), anaplastic lymphoma kinase (ALK), ROS‐1, RET, c‐Met, and neurotrophic tropomyosin receptor kinase (NTRK) 1–3 alterations, are rare [52]. In the case of c‐Met (1–4%; [54, 55]), FGFR3 (3%; [52, 55, 56]), and NTRK1‐3 (1–2%; [55, 57]), alterations occur mostly in the form of gene fusions, which are also found for EGFR in 6–13% of patients [52, 55, 57].
2. Downstream signal transduction cascades induced by RTK activation also frequently bear alterations. This particularly applies to the PI3K/PTEN/AKT/mTOR (phosphatidylinositol 3‐kinase/phosphatase and tensin homolog/AKT/mammalian target of rapamycin) pathway. Taking RTK, PI3K (25–30%, mainly PIK3CA or PIK3R1 alterations), and PTEN alterations (40%) together, at least one of these alterations is found in 90% of GBM [52]. Neurofibromin 1 (NF1) mutations activating the PI3K pathway by reducing its RAS‐inhibiting effect have been found in 10% of cases [52].
3. Genes encoding cell cycle proteins are frequently altered, such as cyclin‐dependent kinase inhibitor 2A/B (CDKN2A/B) deletion (60%) controlling cyclin‐dependent kinase 4/6 (CDK4/6), p53 mutation (20–25%), p53‐inhibiting amplifications of mouse double minute 2 homolog (MDM2) and MDM4 (15%), and RB1 mutation or deletion (8%; mutually exclusive with CDKN2A deletion). At least one of these genes is altered in 90% of GBM [52, 58].
4. Further alterations include DNA repair mechanisms such as mismatch repair deficiency in about 10% of progressive GBM, mostly due to MSH6 loss, and alterations of homologous repair deficiency, DNA checkpoint, and base excision repair [49, 59, 60, 61].

Illustrating the growing drug development pipeline in GBM, all of the aforementioned targets or their associated pathways are currently being investigated as potential treatment options [62, 63]. Despite this well characterized landscape of actionable treatment targets, the identification of successful targeted treatments for GBM remains challenging. In contrast to IDH mutant astrocytoma, where IDH mutation is thought to occur early in gliomagenesis, there is no known early – and thus major – single‐driver alteration in GBM [46, 64, 65]. Further, GBM displays significant cellular and spatial heterogeneity, and potential targets may not be present in most tumor cells [50, 66]. The possibility of longitudinal heterogeneity represents another challenge; it was suggested that molecular targets profoundly change between the newly diagnosed and progressive disease [67, 68], whereas prominent publications reported no substantial longitudinal changes in genetic alteration profiles [51, 69, 70]. Besides the genetic profile, expression patterns of GBM (e.g. EGFR expression) and, in particular, cells of the tumor microenvironment may substantially change over time and have a vast influence on the composition of the tumor tissue and amenability to therapy, further introducing complexity [51].

## 3 Targeted treatment in untargeted study populations

### 3.1 GBM trials with targeted agents

In the last 20 years, targeted drugs have been applied to GBM patients without individual prior target verification, and they have been mostly explored in patients with progressive or recurring tumors following one or more treatment lines. Table 1 provides an overview of drugs, targets, and observed outcomes, focusing on GBM hallmark alterations such as EGFR, PDGFR, FGFR, c‐MET, and the PIK3CA/Akt/mTOR pathway (see also Fig. 1). Beyond this, many drugs directed at less GBM‐specific targets involved in tumor cell growth and/or homeostasis have been evaluated, e.g. transforming growth factor β (TGFβ)‐directed galunisertib [71], CD95 ligand (CD95L)‐directed APG101 [72], Src‐directed dasatinib [73, 74, 75], phosphorylated signal transducer and activator of transcription 3 (pSTAT3)‐directed therapy [76], hepatocyte growth factor/scatter factor (HGF/SF)‐directed rilotumumab [77], and proteasome inhibitors such as marizomib [78]. In addition, there have been several approaches to target receptors thought to be mostly expressed in tumor cells (without individual confirmation beforehand) with locally applied ligand‐toxin fusion proteins targeting, e.g. interleukin 13‐receptor (IL13R), interleukin 4‐receptor (IL4R), or transferrin receptors [79, 80, 81].

**Table 1:** Selection of clinical trials with targeted therapy in adult GBM without molecular pretesting.

Target	Drug	Trials	Major outcomes	Comments	Ref.
EGFR overexpression EGFR amplification EGFR mut EGFR vIII mut	Erlotinib	4 Phase (I)/II, (1 rand., *n* = 110)	rGBM: ORR 3.7–8.4%, PFS6 ≤ 20%, mOS 7.3–9.7 months First line (+RT/TMZ): mOS 19.3 months	No survival signal in rand. trial No impact on downstream signaling in PD/PK trial	[110, 185, 186, 187]
	Gefitinib	3 Phase (I)/rand. II	rGBM PFS6 13%, ORR 0%, mOS 9 months flGBM mOS 11.5 months	Insufficient activity Target engagement but no impact on downstream signaling	[109, 188, 189]
	Lapatinib	2 Phase II	rGBM: PF6 3–17.6%, ORR 0%	No sufficient activity, tumor concentration 497 nm Target engagement but no impact on downstram signaling	[190, 191]
	Neratinib	Phase II rand.	flGBM: mPFS 6.0 (vs. 4.7) months, mOS 13.8 (vs. 14.7 months)	No significant survival effect; in subgroup analysis of EGFR‐activated patients mPFS prolonged (6.3 vs. 4.6 months, *P* = 0.04) but no OS effect	[113, 192]
	Afatinib (Afa)	Phase II rand. (Afa vs. TMZ vs. TMZ/Afa)	rGBM: ORR, mPFS, mOS in Afa arms not superior to TMZmono	No sign of activity	[193]
	Nimotuzumab	Phase III rand.^a^ (*n* = 142)	ndGBM: no significant difference for PFS12, mPFS and mOS	No sign of activity	[194]
	Cetuximab	Phase II (*n* = 55)	Relapsed high‐grade glioma, mPFS 1.8–1.9 months, mOS 4.8–5 months	Limited activity, no correlation of survival with EGFR status	[195]
PDGFR overexpression PDGFR amplifcation	Imatinib	Phase II rand, +/− hypofract RT (*n* = 59)	ndGBM/rGBM: mPFS < 3 months, mOS 5–6.5 months	No measurably activity	[196]
		Phase II (*n* = 231, all + Hydroxyurea; 1st relapse)	rGBM, PFS6 11%, mOS 20 weeks	No clinically meaningful activity	[197]
ALK/ROS1	Ceritinib	Phase 0 (*n* = 7)	rGBM, low drug levels without target engagement	CNS drug levels insufficient for target inhibition	[198]
FGFR	Nintedanib	Phase II (*n* = 25)	No CR/PR, mPFS 1 months	No clinically relevant activity	[199]
c‐MET	Cabozantinib (also VEGFR2 inhibitor)	Phase II (*n* = 152) no prev. anti‐VEGF‐treatment (*n* = 70 with antiangio ther.)	ORR 17.6%	Statistical target not met	[200]
			ORR 4.3%	Modest activity	[201]
	Crizotinib (also ALK/ROS1 inhibitor)	Phase Ib (*n* = 38 first‐line)	mPFS 10.7 months mOS 22.7 months	Regarded as promising, but CNS penetration inferior to other ALK inhibitors	[202]
mTOR	Temsirolimus	Phase II rand vs. TMZ (*n* = 257 *MGMT* unmethylated)	OS1y 72% vs 70% (ns)	Phospho‐mTORSer2448 detected in 38%, associated with temsirolimus benefit	[44]
	Everolimus	Phase II rand, primary (171 RT +/− Ever)	ndGBM. mPFS similar, mOS worse (16.5 vs. 21.2 months)	No sign of activity	[203]
PIK3CA	Paxalisib	Phase II (*n* = 30)	rGBM, mPFS 8.4 months, mOS 15.7 months	Encouraging survival	[204]
	Buparlisib	Phase II (*n* = 15)	PFS6 8%, mPFS 1.7 months	Insufficient activity^b^, incomplete pathway blockade despite BBB penetration	[205]
PARP	Veliparib	Phase II, rando 2:1 (*n* = 125)	ndGBM, mOS 12.7 vs. 12.8 months	Insufficient clinical benefit	[206]
SMO	ABTC‐0904	Phase 0/II (*n* = 40)	PFS6 2.4% mPFS 2.3 months mOS 7.8 months	Insufficient activity^c^	[207]

^a^ Additional randomized trial comprising mainly anaplastic astrocytoma [208].

^b^ Similar lack of activity in combination with chemotherapy [209].

^c^ Significantly more CD133‐producing neurospheres from tumors after ABTC‐0904 than from tumors of untreated patients.

In summary, these approaches failed to achieve convincing results in adult malignant glioma cohorts. Still, some trials performed *post‐hoc* secondary explorative analyses to identify biomarkers for treatment benefit [44, 72], thus justifying the use of the mTOR inhibitor temsirolimus in the ongoing N2M2 trial and indicating a PFS‐prolonging effect of the CD95L‐inhibitor APG101 [72, 82]. In retrospect, the failure of trials without prior target verification, and therefore without enrichment of tumors harboring the targeted alteration, comes as no surprise. Thus, targeted agents must be tested in cohorts preselected for the presence of the targeted genetic alterations.

The only exceptions are multi‐kinase inhibitors with a broad spectrum of therapeutic targets, which enable studies in unselected cohorts. Regorafenib, a multi‐kinase inhibitor targeting VEGFR1‐3, TIE2, PDGFR‐β, FGFR, KIT, RET, and RAF, increased OS in the progressive setting in the randomized phase II REGOMA trial [37]. While questions remain as to the extent to which these positive results rely on the VEGF‐directed antiangiogenic effect [34, 42] and the results have recently been challenged [83], some markers of therapeutic benefit have emerged in explorative analyses. More specifically, the occurrence of a hand‐foot reaction, a common side effect observed in approx. 30% of patients receiving regorafenib, was associated with an increased OS of 6.7 versus 2.6 months in a small retrospective bicentric cohort of patients with progressive glioblastoma receiving regorafenib, and a biomarker analysis of the REGOMA trial described the expression levels of several mRNAs and miRNAs to be associated with survival [84, 85].

## 4 Targeted treatment with previous target verification

### 4.1 Successful trials with molecularly matched drugs in glioma patients

There are a few success stories emphasizing that targeted therapy may show efficacy in molecularly selected glioma subpopulations. In patients with tuberous sclerosis, treatment with the mTOR inhibitor everolimus for subependymal giant cell astrocytomas with alterations in the mTOR pathway is well established and leads to tumor reduction of ≥ 30% in 75% of patients [86]. More recently, the IDH inhibitor vorasidenib increased PFS from 11.1 to 27.7 months in IDH‐mutant grade 2 glioma and allowed for significantly delayed further interventions (likelihood of next treatment intervention or death by 24 months, 16.6% vs. 73%) [87].

In GBM, the only successful molecularly matched treatment to date is combined BRAF/MEK inhibition in patients with a constitutively activated MAPK pathway due to a BRAF V600E mutation. An interim analysis of the single‐arm phase 2 ROAR basket trial exploring this approach in BRAF V600E‐mutated progressive GBM with the BRAF inhibitor dabrafenib and the MEK1/2 (the downstream target of BRAF) inhibitor trametinib showed an objective response rate (ORR, complete or partial response according to RANO criteria) of 32% and a PFS of 2.8 and an OS of 13.7 months [24]. Consequently, EANO guidelines conclude that the clinical benefit in patients with BRAF V600E mutant progressive CNS tumors is sufficiently well established to consider it part of the standard of care [88].

### 4.2 Entity‐agnostic drug registrations for patients with NTRK gene fusions or microsatellite instability/mismatch repair deficiency

While biomarker‐specific drugs are usually marketed for specific cancer types, two drugs received an entity‐agnostic registration: larotrectinib, a tropomyosin kinase receptor inhibitor for tumors bearing NTRK alterations, and the programmed cell death protein 1 (PD‐1) inhibitor pembrolizumab for tumors with microsatellite instability/mismatch repair deficiency. Data are accumulating that in childhood gliomas, larotrectinib addressing NTRK alterations (mostly NTRK fusion transcripts) may induce a high rate of responses [89], but reports on adult GBM patients remain anecdotal, with the largest series reporting disease stabilization (> 6 months) in 4 of 6 patients (Table 2) [90]. A larger series will hopefully provide more reliable information on the efficacy of larotrectinib in this setting. The case is even more challenging for pembrolizumab in patients with microsatellite instability/mismatch repair‐deficient tumors. There are currently no strong data supporting this concept in GBM, as the clinical trial supporting pembrolizumab treatment did not include any GBM patients [91]. Of note, untargeted PD‐1 immune checkpoint inhibition with nivolumab has been extensively studied in newly diagnosed glioblastoma, but failed to prolong survival in large phase 3 trials in *MGMT*‐unmethylated as well as *MGMT*‐methylated newly diagnosed GBM patients [41, 92]. Also, the trial investigating nivolumab at the first relapse of GBM did not show any survival prolongation [93].

**Table 2:** Trials and case series with targeted therapy in molecularly pretested cohorts of GBM and high‐grade glioma.

Drug	Required target	Trial/case series	Major outcome	Ref.
Dabrafenib/Trametinib	BRAF V600E	Phase II, *n* = 45 high‐grade glioma (31 GBM)	12/45 PR 3/45 CR	[24]
Larotrectinib	NTRK gene fusions	*n* = 33 glioma (6 high‐grade adult glioma)	4/6 with some tumor shrinkage 4/6 with treatment duration of 6 months or more (2/6 > 9 months)	[90]
*EGFR‐TKIs* Dacomitinib	EGFR amp	Phase II trial, *n* = 49 rGBM (incl. 19 with EGFRvIII)	EGFRamp only: ORR 6.6%, PFS6 13.3%, mOS 7.8 months EGFRamp + EGFRvIII: ORR 5.3%, PFS6 5.9%, mOS 6.7 months	[210]
		Phase II trial, *n* = 56 rGBM, BEV naive	ORR 3%, PFS6 17%, mOS 10 months	[211]
Osimertinib	EGFR amp, p53wt	Phase II, *n* = 12 rGBM	ORR 0%, PFS6 0%, mOS 5.5 months	[210]^a^
EGFR biologicals (selection) Depatuxizumab mafadotin (ABT414)	EGFR amp	Phase II, *n* = 260, rGBM, rand: TMZ, ABT414, TMZ + ABT414	TMZ + ABT414 vs. TMZ HR 0.71, *P* = 0.06; ABT414 vs. TMZ HR 1.0, *P* = 0.83	[128]
		Phase III, *n* = 639 rGBM, TMZ + ABT414 vs. TMZ + placebo	No improvement of OS (HR 1.02; *P* = 0.63); PFS improved with ABT414 (8 vs. 6.3 months, *P* = 0.029)	[94]^b^
EGFRvIII CAR T	EGFRvIII	Phase I, *n* = 10 rGBM	mOS ~ 7.1 months	[212]
		Phase I, *n* = 18 rGBM		[213]
		Phase II, rand, *n* = 73 rGBM, BEV +/− CDX110	PFS6 28% vs. 16% (*P* = 0.12)	[214]
Rindopepimut (EGFRvIII vaccine CDX‐110)	EGFRvIII	Phase III, rand, *n* = 745 ndGBM, Standard +/− CDX110	mOS 20.1 vs. 20.0 months	[95]
Palbociclib	CDK4/6, RB1 proficiency	Phase II, *n* = 22 rGBM	mPFS 5.1 weeks, mOS 15.4 weeks; stopped for futility	[116]
Capmatinib	PTEN loss/mut + c‐Met amplification (FISH)	Phase II, *n* = 10 rGBM	No response; 3/10 stable disease for 16–20 weeks, stopped for futility	[215]

^a^ Additional single cases and a retrospective case series of *n* = 15 rGBM treated with osimertinib and bevacizumab; pPFS 5.1 months; mOS 9 months; efficacy similar to BEVmono [216, 217].

^b^ In patients with EGFRvIII mutant (HR 0.72, *P* = 0.002) or *MGMT‐*unmethylated tumors (HR 0.77, *P* = 0.012), PFS may be prolonged with ABT414, but no OS improvement.

### 4.3 Lessons learned from unsuccessful trials with molecularly matched drugs

Further trials evaluating targeted treatments in GBM patients with target verification have shown no convincing efficacy data so far (Table 2). This applies to negative data in randomized phase 3 trials investigating depatuxizumab mafodotin, an antibody‐drug conjugate composed of an anti‐EGFR antibody conjugated to a tubulin inhibitor, which found no OS improvement in newly diagnosed GBM with confirmed EGFR‐amplification (18.9 vs. 18.7 months, PFS 8.0 vs. 6.3 months), or rindopepimut, an EGFRvIII‐specific peptide vaccine, which showed no benefit in newly diagnosed EGFRvIII‐positive GBM (OS 20.1 vs. 20.0 months, PFS 7.1 vs. 5.6 months) [94, 95]. For single‐arm trials, an ORR of 25%, which translates to a median OS of 15 months [96], or surpassing a 6‐month PFS rate of 16–20% as observed with CCNU [34, 97], is generally expected for an effective second‐ or later‐line treatment. Examples of single‐arm trials not reaching this threshold are given in Table 2 and include targeting of EGFR, CDK4/6, and amplified c‐MET.

The multifaceted problem of achieving efficacy in trials with molecularly matched treatments is highlighted by the largely unsuccessful therapy of EGFR‐altered GBM despite previous target identification (EGFR amplification in 54%; [98]), which has taught many lessons in this regard:

1. Successful EGFR‐directed therapy in NSCLC is applied in the context of mutations in the tyrosine kinase domain, which activate the receptor. In contrast, EGFR alterations in GBM mainly affect the extracellular domain, and multiple different oncogenic EGFR variants (mostly deletions and missense mutations) typically coexist and are not homogenously distributed [26]. Mechanistically, EGFR alterations in GBM seem to alter ligand discrimination [99], suggesting alternative mechanisms in response to EGFR‐directed therapy in GBM compared to NSCLC (for review, see [26]).
2. EGFR alterations in GBM may not confer oncologic addiction, as they are considered late events in gliomagenesis and are subclonal rather than clonal [100, 101, 102]. This implies that the tumor is a mosaic of cells with different RTK alterations that may cooperate synergistically [103], increasing cellular fitness and resistance to therapy [104]. The resulting spatial heterogeneity renders biopsy sampling less reliable. Further, it implies temporal heterogeneity during further tumor growth, where relapsed GBM may show a loss of mutated targets [67, 105]. This would require target verification in the progressive tumor rather than based on tissue from the primary surgery. While EGFR amplification is usually conserved [106], some trials have shown that staining of the EGFR extracellular domain is changed or lost upon therapy and is not associated with a clinically relevant survival prolongation [95].
3. The treatment of GBM with at least some part of the tumor behind an intact blood–brain barrier represents a pharmacokinetic challenge, as many compounds may not sufficiently penetrate CNS tumors and have a reduced bioavailability, even in the case of in principle sufficient CNS penetration of, e.g., the EGFR inhibitors erlotinib or osimertinib [107, 108]. This contributes to the observation that tumor tissue obtained after EGFR‐directed therapy does not show sufficient target engagement and pathway alterations [109, 110].

The difficulties of EGFR‐directed therapies have been so substantial that new approaches for EGFR targeting, such as EGFR‐directed chimeric antigen receptor (CAR) T cell therapy, are met with caution.

The increasing number of available targeted drugs leads to the question of how to effectively scan for potentially successful target/drug combinations in GBM. Adaptive phase II trials in newly diagnosed *MGMT*‐unmethylated GBM are promising tools to identify drugs for further analysis in confirmatory phase III trials. Examples of such trials are N2M2, INSIGhT, and the Adaptive Global Innovative Learning Environment for Glioblastoma (GBM AGILE), which investigate several targeted drugs in parallel with obligatory molecular testing and a common temozolomide standard arm [82, 111, 112]. These trials are even more intriguing because their results are continuously monitored, and a Bayesian approach is applied to guide the allocation of patients to more successful trial arms. The first results of the INSIGhT trial have already been published, showing superior PFS but similar OS for the CDK4/6 inhibitor abemaciclib (OS 15.3 vs. 14.8 months, PFS 6.2. vs. 4.7 months) and the EGFR/HER2 inhibitor neratinib (OS 14.2 vs. 14.8 months, PFS 6.0 vs. 4.7 months), both in addition to standard radiochemotherapy [113]. However, preliminary results of GBM AGILE challenge the benefit of regorafenib, as mentioned above [83].

## 5 Experience with NGS screening and matched targeted therapy

### 5.1 Important points to consider for NGS‐based individual GBM therapy

Instead of testing single or few drugs in cohorts selected by screening for a single molecular alteration, next‐generation sequencing (NGS) yields an array of genetic alterations for each patient, which may allow the selection of the most promising genetic alteration/targeted drug combination (matched therapy). This approach is an attractive way to evaluate the concept of precision oncology in GBM therapy. In cancer entity‐agnostic case series of patients with metastatic cancer (without GBM patients) such as IMPACT [114, 115], the ORR, 6‐month stabilization rate, median PFS and OS, and 10‐year survival rate of patients receiving matched therapy tended to be higher than those of patients receiving nonmatched therapies. The first steps are made to implement the approach for primary brain tumors in general and gliomas in particular (Table 3).

**Table 3:** Case series with targeted therapy based on NGS screening.

Series	Approach	Therapy decision criteria	Outcome parameters	Results/Signs of activity	Comment
Lazaridis et al. [126]	Retrospective, mostly 2nd relapse *n* = 41 gliomas screened, 36/41 with actionable target, 18/36 with targeted therapy	NGS panel, IHC panel, Seq (TERT), FISH (CCDKN2A/B) Known BBB penetration Molecular tumor board decision	PFS/OS of 16 rGBM with vs. 16 without targeted therapy	mPFS 3.8 vs. 2.0 months PFS6 25 vs. 20% mOS 13 vs. 4 months (*P* < 0.05) High PFS > 7 months and PFS2‐PFS1 > 5 months in Dabrafenib/trametinib (*n* = 2) Cabozantinib (*n* = 2)	Reproduced the known activity of BRAF inhibitors Median 1 prior treatment Precision therapy aspect of Cabozantinib difficult to analyze due to the multikinase activity including VEGFR inhibition
Renovanz et al. [122]	Prospective *n* = 262 GBM screened, 41 treated based on NGS results, 36 evaluable for response	NGS panel, RNA seq, IHC panel Biomarker‐guided ranked molecular tumor board decision	PFS2/PFS1 ratio ≥ 1.3 MCBS score	mPFS 2.5 months 13/36 with PFS2/PFS1 > 1.3 3/6 mTOR‐pathway/everolimus 3/4 FGFR‐TACC fusion/erdafitinib 3/4 FLT missense or EGFR amp/regorafenib 1/5 EGFR/afatinib 1/5 TMB/nivolumab 2/8 EGFR amp + missense/ABT414 MCBS score 1 (PFS ≥ 6 months, or ORR > 60% or ORR 20–60% and DoR ≥ 9 months) in *n* = 5 (2 × Everolimus, 2 × Erdafitinib, 1 × ABT414)	Some signs of activity with FGFR‐fusion or mTOR‐directed therapy > 2 lines of prior treatment Large cohort, also reporting on other CNS tumor entities
Blumenthal et al. [124]	Retrospective *n* = 43 (GBM, AA), 41/43 with actionable targets, 13/43 treated	NGS panel Treatment decision by physician's choice	Response, duration of treatment, mOS	no response with EGFR, MEK, CDK4/6, or mTOR‐directed therapy mOS 4.6 months, longest OS 10 months (EGFR‐directed afatinib)	No clear signs of activity, even with combination treatment Median 1 prior treatment Small and heterogenous cohort
Byron et al. [125]	Prospective 16/20 patients analyzed 13/16 with IDHwt GBM	WES RNA seq IHC panel Molecular tumor board decision	PFS, feasibility: molecularly based treatment decision within 35 days	15/16 recommendations within time frame 7/16 received recommended therapy PFS ~ 3 months *n* = 1 GBM with PFS > 12 months; PFS2/PFS1 = 3.7: Olaparib, trametinib and carboplatin	Feasibility of approach shown Sign of activity in a single GBM patient
Padovan et al. [123]	Retrospective *N* = 417 GBM screened, 343 with actionable targets, 36 with targeted therapy	NGS panel Clinical trials or off‐label compassionate use, decision criteria not reported	PFS2/PFS1 ratio ≥ 1.3 Objective response rate (PR + CR)	mPFS 2.1 months 7/36 with PFS2/PFS1 > 1.3 4/9 dabrafenib/trametinib 2/4 FGFR3/erdafitinib 1/1 MET/capmatinib 0/1 ROS1/entrectinib 3 objective responses (2 dabrafenib/trametinib, 1 entrectinib)	Largest GBM cohort Reproduced known activity of BRAF inhibitors Starting form second‐line treatment Median of targeted therapy lines was 3

To extend this approach to GBM, three major problems have to be addressed:

1. Target identification. In GBM, it is not trivial to infer the most promising target constellation from a list of genetic alterations and whether single or combined alterations represent the best target. For example, it is unknown whether CDK4/6 inhibitors such as palbociclib or abemaciclib can be employed for RB1‐proficient GBM or if CDKN2A/B and CDK4/6 status also need to be considered [116]. Dysregulation of the CDK4/6‐p16‐RB1 pathway is a hallmark of glioblastoma [52]. While CDK4/6 activation inhibits the tumor suppressor protein RB1, allowing cell cycle progression, CDK4/6 inhibitors cause reduced RB1 phosphorylation and apoptosis. Homozygous deletion of CDKN2A/B, encoding the CDK4/6 inhibitor p16, leads to CDK4/6 disinhibition, which might be required for sensitivity to pharmacological CDK4/6 inhibition [116]. CDK4 alterations or RB1 mutations were associated with resistance to CDK4/6 inhibition in patient‐derived GBM xenografts [117]. Similarly, should application of EGFR block in patients with EGFR amplification or activating mutations be given on the base PTEN alterations, which are frequent in GBM and linked to reduced responsiveness to EGFR inhibitors [110]? Clear guidelines for these decisions are lacking. Of note, combined target selection inevitably narrows down treatment options for individual patients.
2. Treatment selection for precision oncology is challenging, irrespective of cancer type. In GBM, this problem may be accentuated as there has only been one successful molecularly guided trial thus far [24]. Therefore, treatment selection mostly has to rely on results from other tumor entities, e.g. breast cancer for DNA damage repair alterations, cholangiocarcinoma and urothelial carcinoma for FGFR alterations, and NSCLC for EGFR alterations [5]. However, applicability may be reduced due to differences in mutation sites, activation of compensatory pathways, and tissue penetration. Treatment selection based on preclinical *in vitro*/*in vivo* results or on biological rationale is even less convincing and leads to a lower strength of recommendation according to current grading guidance [5, 118]. In summary, the paucity of data requires the adoption of treatment strategies based on other tumor entities or preclinical data, both with reduced applicability for GBM.
3. Efficacy assessment. The assessment of treatment success by standard metrics such as ORR, PFS (both largely based on imaging parameters), or OS rates is limited in heterogeneous cohorts of GBM patients receiving precision oncology treatment. Further, interindividual comparison is impaired by the potential prognostic effect of the targeted molecular alterations and the treatment of patients at differing stages of their illness. Intraindividual comparison of PFS until first progression to PFS under matched therapy (PFS2/PFS1) is an elegant alternative, and a PFS2/PFS1 ratio of 1.3 or higher has been accepted as a marker of effective matched therapy in systemic cancers [119, 120]. Considering the progression time scale with a median PFS of 7 months in newly diagnosed GBM and 2 months in progressive GBM, a PFS2/PFS1 ratio of 1.3 translates to a significant PFS increment if the evaluated treatment is initiated at first recurrence and to a numerically small PFS increment if initiated at further recurrence [30]. Accordingly, modifications have been suggested to adjust for very short PFS1 < 2 months to prevent overcalling and for significant PFS2 > 6 months to prevent undercalling of treatment responses in brain tumors, and the concept has been applied in brain tumors [121, 122, 123]. Further approaches include an adaptation of the ESMO Magnitude in Clinical Benefit Scale to brain tumors, combining PFS with imaging response duration (Neuro‐MCBS, see Ref. [122]).

### 5.2 Case series with NGS‐based molecular‐guided GBM therapy in clinical routine

An increasing number of neuro‐oncology centers offer NGS screening for actionable mutations to patients with progressive glioblastoma with no further standard treatment options. At these institutions, multidisciplinary molecular tumor boards are established, providing personalized recommendations for targeted therapies based on individual NGS results. So far, five publications report on mono‐ or oligoinstitutional experience with NGS‐informed personalized therapy [122, 123, 124, 125, 126]: Blumenthal et al. [124] reported the first retrospective cohort from five tertiary hospitals comprising 43 glioma patients (34 GBM), where a NGS panel detected actionable alterations in 95%, leading to targeted treatment in 30% (10/34), but without any treatment response. Byron et al. [125] performed a prospective monocentric trial to evaluate the feasibility of whole exome sequencing‐informed treatment recommendations within 35 days of surgery. Among 16 GBM patients, a recommendation was possible in 94%, 44% (7/16) received a targeted treatment, and one patient (6%) achieved a treatment response. Lazaridis et al. [126] reported a retrospective monocentric cohort of 41 glioma (32 GBM) patients. Following NGS and further methods of genomic profiling, actionable targets were identified in 76% (24/32) and 16 GBM patients receiving targeted treatment achieved an increased PFS (3.8 vs. 2.0 months) and OS (13 vs. 4 months) compared to 16 GBM patients with unmatched empiric treatment. Renovanz et al. reported on their experience from the Center for Personalized Medicine Tübingen, which has an established certified clinical workflow for personalized medicine in the clinical routine for cancer patients without options for trial participation or further registered treatments. This heterogeneous and heavily pretreated cohort included 262 GBM [122]. Following comprehensive molecular profiling, molecularly instructed treatment recommendations were made in 93% (243/262) and 41 GBM patients were treated accordingly, resulting in a PFS2/PFS1 > 1.3 in 36% (13 of 36 evaluable patients). Padovan et al. [123] reported a retrospective, monocentric cohort of 417 GBM patients receiving NGS screening. While actionable targets were identified in 82%, 36 patients (8.6%) received a targeted treatment, of which 20% (7/36) achieved a PFS2/PFS1 > 1.3.

The five series are described in more detail in Table 3. Of note, only two of these studies were prospectively documented [122, 125], and the number of evaluable patients with targeted therapy per cohort remains low, ranging from < 20 [124, 125] to 36 patients [122, 123, 126]. The largest studies to date also highlight the current problem of precision therapy. As reported by Renovanz et al. [122], among 262 GBM patients receiving NGS screening, only 41 actually started matched therapy (about 14% of patients tested). While a high percentage of these (88%, *n* = 36) could be evaluated for efficacy and demonstrate the determination of the authors, the low rate of initiated therapies emphasizes the many obstacles for molecularly matched therapy, such as the rapid deterioration of patients with progressive GBM and the lack of reimbursement by health insurance companies attributable to insufficient GBM‐specific evidence of efficacy [122]. Padovan et al. [123] reported a similar experience, where only 36 (8.6%) of 417 GBM patients receiving NGS screening were able to initiate matched therapy. No treatment response was observed by Blumenthal et al., while Byron et al. report a single patient receiving a potentially successful treatment of olaparib/trametinib/carboplatin with a PFS2/PFS1 ratio of > 3 [124, 125]. The larger studies provide some data on the efficacy of matched therapy in GBM [122, 123, 126].

Lazaridis et al. [126], also considering CNS drug penetration in the decision‐making process, presented 16 patients receiving matched therapy compared to 16 patients with unmatched therapy (Table 3), potentially introducing selection bias. The encouraging PFS results of 3.8 and 2.0 months with versus without matched therapy were mainly driven by the efficacy of BRAF V600E‐directed therapy with dabrafenib/trametinib and c‐Met‐directed therapy with the tyrosine kinase inhibitor (TKI) cabozantinib. While the first observation is in line with the interim results from the ROAR trial discussed in Section 4.1 [24], the cabozantinib results warrant critical discussion as the drug targets not only c‐Met but also VEGFR. Inhibition of the VEGF pathway, e.g. with the VEGF‐A antibody bevacizumab, is well known to prolong PFS but not OS [34, 42, 127]. The intermingling of VEGF‐directed therapy with therapy directed at other targets was also present in the study by Renovanz et al. [122]. As mentioned, 14% of GBM patients started matched therapy, and 13/36 evaluable patients (5% of screened patients or 36% of patients receiving treatment and being evaluable) were successfully treated according to a PFS2/PFS1 ratio > 1.3, while the median PFS was 2.3 months [122]. Here, three of the four patients receiving regorafenib based on FMS‐like tyrosine kinase (FLT) or EGFR alterations did benefit (PFS2/PFS1 > 1.3). As discussed above, regorafenib targets several tyrosine kinases, including VEGFR, raising the possibility that the observed PFS benefit might at least partially be attributable to VEGFR targeting. Apart from this, a signal for treatment benefit was only seen for everolimus in tumors with PTEN or PIK3CA alterations (3/6 with PFS2/PFS1 > 1.3), which is in line with results from the mTOR inhibitor temsirolimus trial in newly diagnosed *MGMT*‐unmethylated GBM [44], and for tumors with FGFR fusion transcripts treated with the FGFR inhibitor erdafitinib (3/4 with PFS2/PFS1 > 1.3). Two patients had a positive PFS2/PFS1 signal with the EGFR antibody‐toxin conjugate depatuxizumab mafodotin in the context of an early‐access program, illustrating its PFS prolongation in a previous trial [128], which did not translate into a longer OS in large phase III trials [94] (Table 3). Padovan et al. [123] confirm these observations: among the 36/417 patients receiving targeted treatment, 19% were treated successfully with a PFS2/PFS1 ratio > 1.3, including three objective responses, again mainly driven by dabrafenib/trametinib in BRAF V600E‐altered GBM and erdafitinib in FGFR3‐altered GBM, while the median PFS was 2.1 months.

Other publications report the results of NGS screening and/or matched therapy allocation without including a substantial number of GBM patients evaluable for treatment efficacy [129, 130]. Using a methodologically different approach, Luger et al. [131] retrospectively analyzed a cohort of 351 patients treated with off‐label therapy and identified 15 patients with high‐grade glioma (8 GBM) who received matched therapy. This series was dominated by the observation that 3/6 patients treated with BRAF V600E‐directed therapy had disease stability for 5+ months. In summary, these reports highlight that few NGS‐screened GBM patients receive targeted treatment, and even fewer may benefit from it.

## 6 Advancing GBM precision oncology: beyond tumor cell targets

**Fig. 2:**
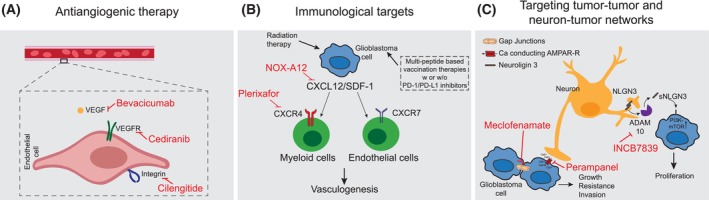
Advancing precision oncology in glioblastoma: selection of potential microenvironmental targets. This figure illustrates potential treatment approaches targeting the tumor microenvironment. (A) Antiangiogenic therapy targeting the tumor‐vascularization axis by vascular endothelial growth factor (VEGF) or integrin inhibition failed in unselected cohorts, but its potential in a precision oncology approach remains promising. (B) Immunological treatment strategies such as personalized vaccination using an individual peptide mix based on tumor tissue analysis have shown sustained immunological responses. Exclusion of myeloid cells by targeting the CXC chemokine ligand 12 (CXCL12)/CXC chemokine receptor 4 (CXCR4) axis reduces postirradiation tumor revascularization and is evaluated in ongoing clinical trials. (C) The disruption of tumor cell networks by gap junction inhibitors might increase radiochemotherapy sensitivity. Neuron‐tumor signaling might be targeted by inhibition of α‐amino‐3‐hydroxy‐5‐methyl‐4‐isoxazoleproprionic acid receptor (AMPAR)‐mediated synaptic input or by ADAMS10 sheddase inhibition to decrease neuroligin 3 (NLGN3) signaling, both mediators of activity‐induced glioma proliferation. For a detailed explanation, refer to Section 6 in the main text. CXCR7, CXC chemokine receptor 7; PD‐1, programmed cell death protein 1; PD‐L1, programmed death‐ligand 1; sNLGN3, soluble NLGN3; VEGFR, VEGF receptor.

While precision oncology heralds potentially great benefit in GBM, the clinical results achieved with targeted drugs remain underwhelming. There are several areas of ongoing research to overcome this. This includes (a) the identification of treatment targets beyond DNA sequencing, such as multi‐omics‐based exploitation of altered pathways, immunophenotyping, epigenetic profiling, metabolomics, and single‐cell analyses [1, 132, 133]. Another area of current research focuses on (b) optimization of the method of target engagement, e.g. including CAR T‐ or NK‐cells, tumor vaccination, oncolytic viruses, and antibody‐drug conjugates (see [134] for review), despite the low frequency of currently addressable targets [135, 136, 137]. (c) The optimization of (sequential) target verification, e.g. via radiomic and liquid biopsy strategies [1, 138, 139], could also improve the extent of the therapeutic benefit/treatment response. (d) Understanding and overcoming molecular mechanisms of acquired therapy resistance is key to developing more potent therapeutic modalities [140], and (e) the optimization of clinical trial conductance could better inform future studies [141, 142]. Here, we focus on discussing the advancement of precision oncology in GBM toward novel treatment targets beyond tumor cell‐intrinsic targets, thus including targets in the tumor‐associated microenvironment (TME). The TME of GBM is increasingly well characterized [143], and changes in the TME may be crucial for tumor progression [51]. Interactions of tumor cells with vascular structures, immune cells, neurons, glial cells, and each other may provide new targets for therapy (Fig. 2).

In this context, extracellular vesicles (EV) are notable for their involvement at both a diagnostic and therapeutic level. EVs are membrane‐bound vesicles secreted into the extracellular space that can cross the blood–brain barrier and carry a broad range of cargos, including nucleic acids, lipids, and proteins, together with markers reflecting their biogenesis [144]. GBM‐derived EVs purified from blood or cerebrospinal fluid allow for tumor diagnosis and noninvasive longitudinal sampling for detection of tumor progression, treatment targets, and treatment response [144, 145, 146, 147, 148, 149]. Further, EVs may be taken up by neighboring and distant cells in the TME as well as GBM cells, thus representing both an important means of GBM‐TME communication and a possible therapeutic approach for targeted drug delivery [150, 151, 152, 153].

### 6.1 Antiangiogenic therapy

The tumor‐vascularization axis has been targeted with the application of the VEGF‐A inhibitor bevacizumab, leading to prolonged PFS (potentially also due to antiedematous effects) but not OS, both in newly diagnosed and progressive GBM [34, 42, 127, 154]. Other anti‐angiogenic drugs, such as cediranib, a VEGFR inhibitor, had a similar PFS benefit in newly diagnosed GBM, but again, there was no OS benefit both in the newly diagnosed and progressive settings [155, 156]. Cilengitide, an integrin inhibitor targeting angiogenesis, did not find its way into clinical application following a PFS‐ and OS‐negative phase III trial [40, 157]. However, antiangiogenic therapy was applied in unselected cohorts without pretesting for the respective targeted angiogenic factors, and thus its potential might be higher in a precision oncology approach. Retrospective analyses aimed to identify molecular subgroups with an OS benefit from bevacizumab [158, 159]. In a biomarker analysis of AVAglio, the ‘proneural’ gene expression subtype was associated with a significant OS advantage (17.1 vs. 12.8 months) in newly diagnosed GBM receiving bevacizumab, which seems counterintuitive as this subtype is associated with lower VEGF expression, and the results could not be confirmed in the GLARIUS trial [46, 127, 158, 160]. In the progressive setting, NF1 mutation was associated with survival benefit from bevacizumab (OS approx. 17 vs. 8 months) in an exploratory biomarker analysis of EORTC‐26101, but these results need further validation [159].

### 6.2 Immunological targets

The interaction of the immune system with GBM cells may provide further targets for precision oncology. To date, large clinical trials investigating immune checkpoint blockade in GBM have failed [41, 92, 93], and no predictive markers have been defined for matched therapy besides microsatellite instability (see Section 4.2). Alone or in combination with PD‐1/programmed death‐ligand 1 (PD‐L1) inhibitors, some trials applied cell‐based (e.g. DCVax [161]) or – more promisingly – multi‐peptide‐based vaccination therapy [162] after EGFRvIII‐directed mono‐peptide vaccination with rindopepimut failed as discussed above [95]. The peptide vaccination approach may be personalized using an individual peptide mix informed by tumor tissue analysis. First results from the phase I GAPVAC‐101 trial, investigating highly individualized vaccinations against an individual selection of unmutated antigens and neoepitopes in 15 patients, document a sustained T‐cell immune response, while meaningful clinical efficacy (e.g. prolongation of PFS and OS) has still not been demonstrated [163]. It remains a major challenge to identify immunomodulatory targets in the microenvironment that can overcome local immunosuppression and further enhance the immune reaction against tumor cells. Further, macrophages and microglia have been shown to interact with tumor cells and may even manipulate them to obtain a more aggressive phenotype [164]. Despite promising preclinical data, first approaches with colony stimulating factor 1 receptor (CSF‐1R)‐targeted inhibition of macrophages failed [165, 166, 167]. Macrophage exclusion from the tumor by inhibition of C‐X‐C motif chemokine receptor 4 (CXCR4) was shown to reduce post‐irradiation tumor revascularization in a small phase I/II trial. Additionally, inhibition of CXCR4 by plerixafor or inhibition of its ligand C‐X‐C motif chemokine ligand 12 (CXCL12; formerly known as stromal cell derived factor‐1, SDF‐1) by NOX‐A12 is being evaluated in several ongoing trials [168, 169]. Macrophages and microglia provide several other markers that may be targeted to enable a stronger and more precise immune reaction in GBM immunotherapy [170]. Further, indirect targeting of immune cells with GBM‐specific stromal protein‐targeted immunostimulatory cytokines represents a novel approach. An antibody‐cytokine conjugate targeting a tumor‐associated fibronectin epitope to enable local distribution of tumor necrosis factor was associated with increasing tumor necrosis and local inflammation in a phase I study, with objective responses in 3/5 progressive GBM patients, and is currently being evaluated in further trials [171, 172].

### 6.3 Targeting tumor‐tumor and neuron‐tumor networks

The interactions of tumor cells with each other and with neuronal or glial cells offer further opportunities for precision oncology. The rising field of cancer neuroscience has provided a host of landmark publications, showing that GBM form tumor microtube‐based tumor cell networks that confer resistance to radiotherapy and chemotherapy [173, 174, 175, 176] and promote tumor cell invasion by recapitulating developmental neuronal programs [177]. These observations may inform new targets for future therapeutic manipulation, e.g. or the disturbance of hub cells within the syncytium that dominate and organize the tumor cell network [178], or the disruption of tumor syncytia by gap junction inhibitors [174, 179, 180] – the latter being explored in an ongoing phase I/II trial [181].

Finally, several ways to modulate the neuronal input on tumor cell networks have been found and may be targeted. The synaptic protein neuroligin‐3 (NLGN3) was identified as the leading mitogen mediating neuronal activity‐induced glioma proliferation in patient‐derived xenograft models, and reduction of the release of its soluble form (sNLGN3) by ADAMS10 sheddase inhibition with INCB7839 is explored in a phase I trial (NCT04295759) [173, 182]. Similarly, neuronal activity was shown to mediate glioma invasion and growth via α‐amino‐3‐hydroxy‐5‐methyl‐4‐isoxazoleproprionic acid receptor (AMPAR)‐mediated synaptic input from neurogliomal glutamatergic synapses in patient‐derived xenograft models. In line with this, targeting the modulation of AMPAR synaptic transmission using the antiepileptic drug perampanel is explored in a phase II trial [176, 183]. To further refine this as a precision therapy approach, predictive markers have yet to be defined.

## 7 Summary and further perspectives

Despite first successes with BRAF V600E‐directed dabrafenib/trametinib, and signs of some efficacy in a low percentage of GBM patients receiving molecular‐guided therapies, precision oncology has yet to find broad clinical application with proven efficacy in patients with GBM. Trials investigating targeted drugs in molecularly defined subgroups and treatment allocation based on broad NGS screening need further optimization, e.g. by taking into account the CNS penetration of drugs, more complex prediction models based on combinations of genetic vulnerabilities/interaction of pathways [184], and new targets beyond the tumor cell. New models of clinical trials are being conducted to allow efficient analysis of new substances and multi‐omics approaches. The results of N2M2, GBM AGILE, and INSIGhT exploring multiple targeted treatments in comparison to a common standard of care will significantly advance the field. Until more efficacy data are available, matched personalized therapy may, with the exceptions mentioned above, be reserved for the experimental treatment of relapsed GBM.

## Conflict of interest

UH reports advisory and speaker honoraria from Bayer and speaker honoraria from Medac. The other authors report no conflicts of interest.

## Author contributions

UH conceptualized and supervised the project. JW and UH researched the literature and wrote the first draft of the manuscript. A‐LP designed the figures. All authors contributed to the writing and editing of the manuscript.
